# Remote sensing and spatial statistical techniques for modelling *Ommatissus lybicus* (Hemiptera: Tropiduchidae) habitat and population densities

**DOI:** 10.7717/peerj.3752

**Published:** 2017-08-31

**Authors:** Khalifa M. Al-Kindi, Paul Kwan, Nigel R. Andrew, Mitchell Welch

**Affiliations:** 1 School of Science and Technology, University of New England, Armidale, NSW, Australia; 2 Centre for Excellence for Behavioural and Physiological Ecology, University of New England, Armidale, NSW, Australia

**Keywords:** Remote sensing, Dubas bug, *Ommatissus lybicus*, Spatial statistics

## Abstract

In order to understand the distribution and prevalence of *Ommatissus lybicus* (Hemiptera: Tropiduchidae) as well as analyse their current biographical patterns and predict their future spread, comprehensive and detailed information on the environmental, climatic, and agricultural practices are essential. The spatial analytical techniques such as Remote Sensing and Spatial Statistics Tools, can help detect and model spatial links and correlations between the presence, absence and density of *O. lybicus* in response to climatic, environmental, and human factors. The main objective of this paper is to review remote sensing and relevant analytical techniques that can be applied in mapping and modelling the habitat and population density of *O. lybicus*. An exhaustive search of related literature revealed that there are very limited studies linking location-based infestation levels of pests like the *O. lybicus* with climatic, environmental, and human practice related variables. This review also highlights the accumulated knowledge and addresses the gaps in this area of research. Furthermore, it makes recommendations for future studies, and gives suggestions on monitoring and surveillance methods in designing both local and regional level integrated pest management strategies of palm tree and other affected cultivated crops.

## Introduction

Remote sensing (RS) is a powerful technology that has been applied in precision agriculture applications (Shah et al., 2013). Remotely sensed data can be used in mapping tools to classify crops and examine their health and viability. They can also be used for monitoring farming practices and to measure soil moisture across a wide area instead of at discrete point locations that are inherent to ground measurement (Atzberger, 2013). Based on these spatial differences, variable rate application of chemicals such as fertilisers or pesticides can be made. Remote sensing information can further be used to establish sub-field management zones, providing a less expensive yet finer resolution option than grid sampling.

Although RS technologies are more widely used in other industries, their potential for profitable use by farmers is less frequently studied. As examples in agriculture, RS technologies have been used successfully for monitoring and mapping water stress, crop quality and growth, wetland, water quality, phosphorus and nitrogen deficiencies in vegetation, as well as detecting and predicting insect infestations (e.g., *O. lybicus*) (Al-Kindi et al., 2017a; Gooshbor et al., 2016; Lamb & Brown, 2001; Riley, 1989) and plant diseases (Neteler et al., 2011).

### Background

The date palm, *Phoenix dactylifera* Linnaeus, is an important economic resource in the Sultanate of Oman. Plant-parasitic nematodes, associated with date palm trees in Oman and in most other Arab countries, can reduce their economic yields (El-Juhany, 2010). A variety of insect pests can cause major damages to this crop through production losses and plant death (Abdullah, Lorca & Jansson, 2010; Al-Khatri, 2004; Blumberg, 2008; El-Shafie, 2012; Howard, 2001). One such species, *Ommatissus lybicus* de Bergevin 1930 (Hemiptera: Tropiduchidae), which is known more commonly as the Dubas bug (DB), has been identified as a major economic threat, and is presently affecting palm growth yield in Oman (Al-Yahyai, 2006). Indeed, the DB has been identified as one of the primary reasons for the decline in date production in Oman (Al-Yahyai & Al-Khanjari, 2008; Al-Zadjali, Abd-Allah & El-Haidari, 2006; Mamoon, Wright & Dobson, 2016). It is also a principal pest of date palms in many locations throughout the Middle East, East and North Africa, (Klein & Venezian, 1985; Mifsud et al., 2010). The DB is believed to have been introduced into the Tigris-Euphrates River Valley, from there it has spread to other zones in recent decades (Blumberg, 2008; El-Haidari, Mohammed & Daoud, 1968).

The DB is a sap feeding insect; both adults and nymphs suck the sap from date palms, thereby causing chlorosis (removal of photosynthetic cells and yellowing of fronds). Prolonged high infestation level will result in the flagging and destruction of palm plantations (Al-Khatri, 2004; Howard, 2001; Hussain, 1963; Mahmoudi et al., 2015; Mokhtar & Al Nabhani, 2010; Shah et al., 2013). There is also an indirect effect whereby honeydew secretions produced by the DB can promote the growth of black sooty mould on the foliage and consequently a reduction in the photosynthetic rates of date palms (Blumberg, 2008; Mokhtar & Al-Mjeini, 1999; Shah et al., 2012). Nymphs pass through five growth instars (Hussain, 1963; Shah et al., 2012), with adult female DB reaching 5–6 mm and the males 3–3.5 mm in length (Aldryhim, 2004; Mokhtar & Al Nabhani, 2010). Their colour is yellowish-green while the main distinguishing feature between males and females is the presence of spots on females; males have a more tapered abdomen and larger wings relative to the abdomen (Al-Azawi, 1986; Al-Mahmooli, Deadman & Al-Wahabi, 2005; Elwan & Al-Tamimi, 1999; Hussein & Ali, 1996; Jasim & Al-Zubaidy, 2010; Kaszab, Wittmer & Buttiker, 1979; Khalaf et al., 2012; Mokhtar & Al Nabhani, 2010; Thacker, Al-Mahmooli & Deadman, 2003).

### Study area

**Figure 1:**
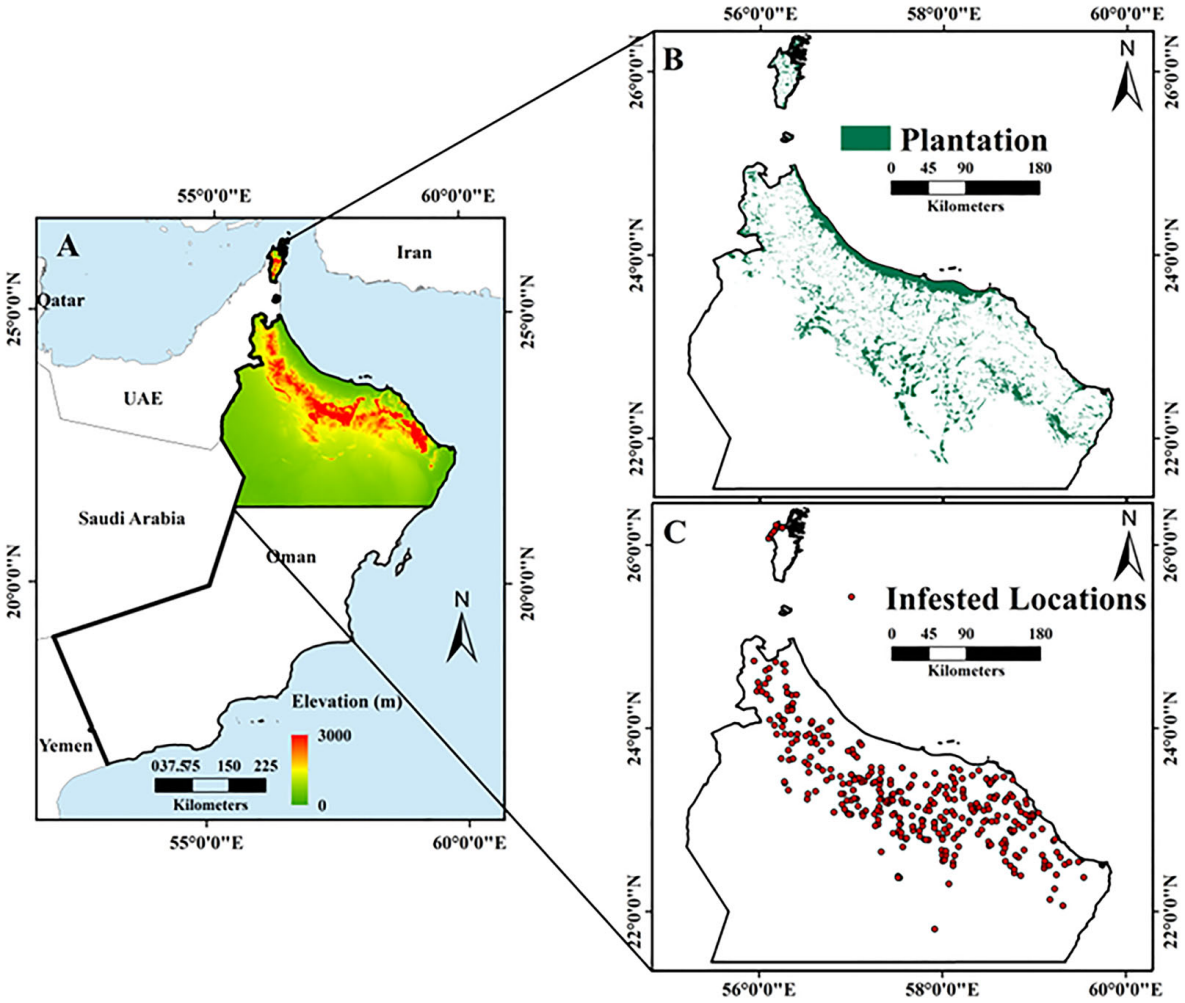
Maps of the study area, including: (A) topography and location of Oman, with the study area outlined by the black rectangle; (B) elevation change within the study area; and (C) distribution of date palm plantations in the study area (Esri ArcGIS 10.3).

The Sultanate of Oman, which covers an area of 309,500 km^2^, extends from 16°40′N to 26°20′N, and 51°50′E to 59°40′E. It occupies the south-eastern corner of the Arabian Peninsula (Fig. 1). It has 3,165 km of coastline, extending from the Strait of Hormuz in the north to the border with the Republic of Yemen in the South. The coastline faces onto three different water bodies, namely the Arabian Sea, the Persian Gulf (also known as Arabian Gulf), and the Gulf of Oman.

To the west, Oman is bordered by the United Arab Emirates and the Kingdom of Saudi Arabia. Mountainous areas account for 15% of the land area, while desert plains and sandy areas cover 74%, agro-biodiversity areas cover 8%, and the coastal zone covers 3%, respectively (Luedeling & Buerkert, 2008). The location of Oman provides favourable conditions for agriculture, with land under agricultural use accounting for 8% of the territory and the economic output accounting for 14.6% of the GDP in 2008. According to the 2004–2005 soil survey conducted by the Ministry of Food and Agricultural (MFA), 22,230 km^2^ (equivalent 2.223 million ha) is optimal for agricultural activity, which represents ∼7.5% of the country’s land area. Approximately 728.2 km^2^ (∼72,820 ha) of the country is irrigated using the Falaj irrigation system, where local springs or wadis (streams) underflow areas are cultivated with palm trees, banana, limes, alfalfa, and vegetables (Gebauer et al., 2007).

Oman has an arid climate, receiving less than 100 mm of rainfall per year; however, the mountainous parts of the country receive higher precipitation levels (Kwarteng, Dorvlo & Vijaya Kumar, 2009). As the dependent variable, DB infestations occur where palm trees are concentrated; therefore, in this study we focused on northern Oman (26°50′N to 22°26′N, and 55°50′E to 59°50E) which experiences high infestations (Fig. 1) (Al-Kindi et al., 2017b).

Dubas bugs are active on leaflets, rachis, fruiting bunches, and spines during different stages of growth of date palm trees. These infestations are capable of causing up to 50% crop loss during a heavy infestation (Shah et al., 2013). Studies of insect pests of the date tree palm indicated more than 54 arthropods species insects connected with date plantations. Nevertheless, DB and red weevil (RPW) *Rhynchophorus ferrugineus* Oliver, and lesser moth, are considered major economically significant pests affecting growth and yield of date palm trees (Al-Zadjali, Abd-Allah & El-Haidari, 2006).

### Biology and life history

The biology of this species has been investigated in a number of studies (Al-Azawi, 1986; Arbabtafti et al., 2014; Hussain, 1963; Jasim & Al-Zubaidy, 2010; Klein & Venezian, 1985; Payandeh & Dehghan, 2011; Shah et al., 2012). The DB produces two generations annually, including the spring and autumn generations (Blumberg, 2008; Hussain, 1963). In the spring generation, eggs start hatching from February to April, after which nymphs pass through five instars to become adults in approximately 6–7 weeks. The eggs aestivate during the hot season (i.e., summer) until the autumn generation, when they start hatching from late August to the last week of October. A nymph takes about 6 weeks to develop into an adult, which then lives for about 12 weeks. Females lay between 100 and 130 eggs (Elwan & Al-Tamimi, 1999; Mokhtar & Al Nabhani, 2010). The female DB lays her eggs by inserting them into holes in the tissue of the date palm frond at the end of each generation. The eggs remain dormant for about three months. When they hatch, the resulting nymphs remain on the fronds of the same tree, feeding on the sap, and defecating large amounts of honeydew, which eventually covers the palm fronds and promotes the growth of black sooty mould (Zamani, Aminaee & Khaniki, 2013).

In extreme cases, the sooty mould that develops from the honeydew secretions can block the stomata of the leaves, causing partial or complete suffocation of the date palm, which in turn reduces its yield. The honeydew secretion also makes the dates unpalatable (Aminaee, Zare & Assari, 2010; El-Juhany, 2010; Gassouma, 2004; Mamoon, Wright & Dobson, 2016). The eggs of DB are sensitive to temperature. In summer, the eggs can hatch within 18–21 days, but in winter they may take up to 170 days to hatch (Blumberg, 2008). The developmental time of DBs eggs has been studied at three different temperatures, 17.6, 27.5, and 32.4 °C in Oman (Al-Khatri, 2011). The results showed that a temperature of 27.5 °C appeared to be the optimal temperature for the biological activities of this species (Al-Khatri, 2011). At 35 °C, the biological processes of the pest are disrupted, thus leading to high mortality, particularly in females (Bagheri et al., 2016; Bedford et al., 2015).

Investigations into the population and the fluctuation in spatial distribution (Khalaf & Khudhair, 2015) of the two DB generations in the Bam region of Iran showed that this pest has an aggregated spatial distribution pattern (Payandeh, Kamali & Fathipour, 2010). Seasonal activities effected by climate change showed that nymphs were dynamic from April to May in the first generation and August to October in the second generation. In the first and second generations, the adults are active from May to June and from September to November, respectively. Earlier work (Blumberg, 2008) reported that temperature exposure below 0 °C adversely affects the ability of adults to survive. The DB avoids direct sunlight (Klein & Venezian, 1985; Shah et al., 2013), and it is spread via the transfer of infested offshoots as well as by wind or wind dust (Hassan, 2014; Jasim & Al-Zubaidy, 2010).

### Biological control

Some research has also been conducted on the natural biological control of the DB, such as using predators and parasites. Early results showed a variety of natural predators that could be used as biological control agents, among these being *Aprostocetus* sp., *Oligosita* sp., and *Runcinia* sp. (Cammell & Knight, 1992). Furthermore, Hussain, 1963 reported that the eggs of the DB could be parasitised by a small Chalcidoid, while the nymphs and adults were preyed upon by the larvae of the lace wing (*Chrysopa carnea Steph.*). Hussain also stated that three adult species of Coccinellids prey on nymphs and adults of the DB. However, further study is needed to determine the distributions of these natural enemies in Oman and their effectiveness in controlling DB populations. Some measure of success was also achieved with pathogenic bacteria as microbiological control agents (Khudhair, Alrubeai & Khalaf, 2016), although their toxicological aspects require further research in order to assess the safety of their implementation at a large scale (Cannon, 1998).

### Chemical control

Given the significant economic impact of this pest, research into effective management strategies demands high priority. Several insecticides have been evaluated for DB control in Oman since 1962 (Table 1) with Sumi-Alpha-5 EC being effective as a ground spray and Karate® 2 ULV, Trebon® 30 ULV, and Sumicombi 50® ULV achieving some measure of success as aerial sprays. Karate-Zeon® was also found to be very effective as it gave 100% reduction in numbers of DB instars and adults, between three and seven days after application. However, the use of this particular pesticide is restricted due to its side effects such as irritation (Al-Yahyai & Khan, 2015). In Israel, systemic carbamates such as aldicarb and butocarboxim have been successful, while in Iraq dichlorvos (DDVP) injected directly into the infected palms were also successful in suppressing the pest population (Blumberg, 2008). Nonetheless, this method of control is expensive with negative environmental impacts on non-target species (particularly natural enemies of DB) as well as on human health.

**Table 1:** Major pesticides used in Dubas bug management in Oman.

Brand names	Active ingredients	Chemical group
Dubaklin	Dintefurn 10% ULV	Neonicotinoid
DECIS	Deltamethrin 12.5% ULV	Synthetic pyrethroid
Sumicombi-Alpha	Fenitothion %24.5 + esfenvalerate %0.5 ULV	Organophosphate + pyrethorid
Trebon	Etofenprox %20 EC	Non-ester pyrethroid
Sumi-Alpha	Esfenvalerate %0.5% EC	Synthetic pyrethroid
Kingbo	Oxymstrin %0.2 & 0.6 SL	Botanical
Actellic	Pirimiphos-methy1 %50 EC	Organophosphate
Pyrethrum	Pyrethrums %50 EC	Botanical
Sumi-Mix	Fenitrothion 25% + fenpropathrin %2.5 EC	Organophosphate + pyrethorid
1-Green	Angulation A: %1 W/V	Botanical
Karate-Zeon	Lambda-cyhalothrin %10 CS	Synthetic pyrethyroids
Fytomax	Azadirachtin %1 ULV	Botanical

Research has shown that some pesticide residues persist in the fruit up to 60 days after application (Al-Samarrie & Akela, 2011). In addition, chemical control has achieved limited successes and DB continues to pose a major challenge to Omani agriculture. More information about the biological and chemical control and their impacts can be found in literature (Shifley et al., 2014; Thacker, Al-Mahmooli & Deadman, 2003).

### Research opportunities

A number of opportunities exist for research into the biology and ecology of this species in order to gain a thorough understanding of its life cycle and its distribution. The climatic factors that influence its survival and distribution also merit study because such information may be useful in determining current and future potential distributions, particularly in light of climate change.

In a review of the effects of climate change on pest populations, an earlier report (Cammell & Knight, 1992) suggested that increases in mean global temperatures, as well as changes in rainfall and wind patterns, could have profound impacts on the population dynamics, abundance, and distribution of insect pests of agricultural crops. More recent research has supported this finding (Bale et al., 2002; Cannon, 1998; Cook, 2008; Shifley et al., 2014; Tobin, Parry & Aukema, 2014). A key issue in ecology and conservation is the mapping of pest species distributions as this information can be used to devise more effective management strategies for their control.

Mapping of DB infestations is important for developing predictive models that give the probability of occurrence, spatial distributions and densities under different environmental, meteorological, anthropogenic and resource availability conditions. Maps such as the DB hazard map can be used to devise an integrated palm management (IPM) plan, thus enhancing capacity and educating farmers on how to apply IPM for the control of this pest.

Mapping DBs are also beneficial in terms of better planning of date palm orchard locations, better design and management of farms, what cultivars to plant, distance between palms, irrigations, pesticides, fertilisations, etc. (Bouyer et al., 2010). There will also be savings in the cost of monitoring since RS based techniques developed as part of this study can provide a more efficient and cost-effective means for large scale monitoring of infestations and observation of stress levels on date palm trees.

The aim of this review is to highlight technological advances in the fields of RS (i.e., by aircraft or a satellite platform) and spatial statistical techniques that can be used to significantly enhance our ability to detect and characterise physical and biological stresses on several plant species. In particular, advanced RS and spatial statistical techniques need to be developed and implemented for the surveillance and control of DB adults and nymphs over large spatial scales. In turn, this will greatly assist plant protection service (PPS) projects, integrated pest management technology (IPMT) programs and farmers in protecting their palm tree orchards by adopting timely preventative actions.

## Remote Sensing Data

### Data requirements for crop management

It is important to collect data regarding crops and soil and to identify the changes that occur in the field to achieve precise crop management in the agricultural sector. Pinter et al. (2003) Data are needed on the conditions that are stable across seasons (e.g., crop type, soil fertility), differing during the seasons (e.g., pest attacks, water quality and quantity, nutrient contents, moisture, temperature), and on factors that contribute to crop yield variability (Hall, Skakun & Arsenault, 2006; Jadhav & Patil, 2014). This data is valuable for determining the unique phenological cycles of agricultural crops in different geographic regions (Jensen, 2000; Abdullah & Umer, 2004; Acharya & Thapa, 2015).

A good example of this are date palms. Typically, date palm trees are 7–10 m tall with crowns 2–4 m in diameter, and the trees are normally spaced 3–5 m apart. The understory of date palm plantations might include banana palms, mango trees, acacia bushes, vegetable crops, grain crops, forage crops. The reflectance characteristics of a date palm area are often driven by the density and health of the understory vegetation (Harris, 2003). It can be difficult to use small pixel data to study date palm areas with little or no understory vegetation because the small pixel effects may make it difficult to identify infestations (e.g., where date palms are infested between mountains and dry rivers) given the tree spacing and density of leaves and branches. Studies like Hussain (1963) and Mahmoudi et al. (2015) have revealed that heavy infestations occur mostly along valleys. Additionally, the characteristics of the understory vegetation may dominate the contribution of spectral responses rather than the tree vegetation themselves.

### Optical remote sensing data

The vital feature of RS is the detection of radiant energy emitted by various objects. The energy detected might be in the form of acoustic energy (sound) or electromagnetic energy (visible light, infrared heat, ultraviolet, and microwaves). Remote sensing technology deployed from ground, air, or space-based platforms is capable of providing detailed spectral, spatial, and temporal information on vegetation health and is particularly useful for crop yield estimation applications (Justice et al., 2002).

#### Temporal resolution of remote sensing data

The temporal resolution of remote sensing data is important for commercial monitoring or management projects. The commercial Landsat and SPOT have revisit intervals of 16 and 26 days, respectively. The IKONOS revisit times range from 1 to 3 days. On the other hand, airborne (aircraft-mounted) sensors are more amenable to user defined re-visitation. The capacity of high temporal resolution RS technology has been exploited for monitoring seasonal vegetation variations, over wide areas is the estimation of net primary production and deciding time boundary conditions for crop yield modelling (Hatfield & Pinter, 1993; Marx et al., 2010; Reynolds, Reynolds & Riley, 2009; Reynolds & Riley, 1997). We believe temporal RS data can be used to study seasonal DB infestations because there are two generations, namely spring and autumn.

Longer term temporal images (e.g., covering a 15-year period) can be used to classify and to determine the directions and speed of spread of DB infestations. This approach can also be applied to historical images to obtain as much information as possible on selected areas. Change detection can also be performed to quantify the degree of variation in the infestation levels that is needed to occur before the change can be detected by satellite technology. This is important for the development of a management and surveillance strategy for DB.

#### Spatial resolution of remote sensing data

Spatial resolution is measured in terms of the smallest target on the ground. The number of available image-forming pixels in the sensor and its distance from the ground contribute to determining the pixel-size on the ground and the overall image footprint allowing low and high spatial resolution data on insect pests like DB (Kerr & Ostrovsky, 2003). Depending on the goals of a study, technology with an appropriate spatial resolution should be chosen. For example, certain Landsat data sets have spatial resolution of 30 m while certain SPOT data sets have spatial resolution of 20 m in each dimension. If it is a large scale study (e.g., large orchard), Landsat imagery at a 30 m resolution may be sufficient (White & Roy, 2015).

However, if the study is for small orchards surrounding the mountains where several types of plantations are present, high resolution data would be needed. High resolution imagery products are available, such as SPOT’s panchromatic 10 m resolution data sets and Landsat’s multispectral scanner 20 m resolution imagery, Wolter, Townsend & Sturtevant (2009). Furthermore, very high resolution imagery are available, including QuickBird’s 2.15 m resolution images or the National Agricultural Imagery Programme’s (NAIP’s) 1m resolution orthophotographs (Boryan et al., 2011).

More recently, high resolution satellite imagery from IKONOS, which consists of 4 m resolution multispectral imagery, have become available; but the costs for obtaining such data remain a significant impediment to their widespread use. These high resolution images can be used to classify and map the spatial distribution and infestation levels of DB. Very high resolution data collected with unmanned aerial vehicle (UAV)-based remote sensing technology can be used for detecting and mapping of plant diseases and infestations such as those due to DB (Colomina & Molina, 2014; Kattenborn et al., 2014; Loper, 1992; Nebiker et al., 2008; Sperlich et al., 2014; Zhang & Kovacs, 2012).

#### Spectral resolution of remote sensing data

Spectral resolution is typically defined as the number of bands of the electromagnetic spectrum that are sensed by the RS device. A very important aspect of spectral resolution is the width of the bands. Different band-widths have been employed extensively in multispectral imagery applications (Zwiggelaar, 1998), and these data often cover an entire colour or colours such as, the red and blue bands of the spectrum. Multispectral systems commonly obtain data for 3–7 bands in a single observation such as in the visible and near-infrared (NIR) regions of the electromagnetic spectrum (dos Santos et al., 2016). Multispectral imagery, however, lacks the sensitivity to detect subtle changes in tree canopy reflectance that are caused by physiologic stress from insects or pathogens (Lawrence & Labus, 2003).

Dakshinamurti et al. (1971) found that multispectral photography is useful for clearly differentiating between coconut plantations and other crops such as jack fruit, mangoes, and bananas in India. Another relevant study, Leckie et al. (2004), used multispectral data for detecting and assessing trees infested with *Phellinus weirii* which causes Laminated root rot disease. Other work (Stephens, Havlicek & Dakshinamurti, 1971) has shown that multispectral photography can be used to clearly distinguish between many types of fruit orchards and crops.

Hyperspectral imagery tends to have much narrower band widths, with several to many bands within a single colour of the spectrum (Jadhav & Patil, 2014). These might include the visible (VIS), NIR, mid-infrared (MIR), and thermal infrared portions. In the visible portion of the electromagnetic spectrum (400–700 nm), the reflectance of healthy green vegetation is relatively low because of the strong absorption of light by the pigments in plant leaves (Apan, Datt & Kelly, 2005; Shafri & Hamdan, 2009; Teke et al., 2013). If there is a reduction in pigments (e.g., chlorophyll) due to pests, the reflectance in the affected spectral region will increase (Carter & Knapp, 2001; Prabhakar et al., 2011). A past study (Vigier et al., 2004) reported that reflectance in the red wavelengths (e.g., 675–685 nm) dominated most of detection data for *Sclerotinia* spp. stem rot infections in soybeans. Over approximately 700–1,300 nm (the NIR portion), the reflectance of healthy vegetation is very high. Damages caused by DB infestations in the form of black sooty mould on palm tree leaves and understory vegetation that is promoted by bug excrement causes overall reflectance in the NIR region to be lower than expected. The new hyperspectral RS technology could be used to develop early (pre-visual) detection methods for DB infestations.

Colour-infrared technology with supporting hyperspectral reflectance data could be used to identify specific trees and fronds of date palm trees that have been infested with DB. These methods can be used to monitor changes in infestation levels according to honeydew, which is converted to sooty mould on the fronds during high levels of infestation. Honeydew secretion is a good indicator of DB feeding activity (Al-Abbasi, 1988). The indirect assessments of the insect populations can be carried out by measuring the amounts of honeydew caused by the insects (Southwood, 1978). Additionally, airborne visible/infrared imaging spectrometer (AVIRIS) can be used to determine the extent and severity of DB infestation damage in different areas.

### Radar data

For many years, airborne technology has been employed in agricultural operations. Nevertheless, space-borne synthetic aperture radar (SAR) technology such as those of the Advanced Land Observing satellite; TerraSAR-X and Phased Array L-band have become available since the 2000s (Ortiz, Breidenbach & Kändler, 2013). Multiple radar sensors can work autonomously to detect solar radiation variation, but dissimilar optical sensors from which spectral reflectance measurements are taken are affected differently by variation in the solar emission. Radar technology has found limited applications in regional studies because of its high costs, the narrow swath widths, and limited extent of coverage (Feng et al., 2003).

The data can be extracted routinely by using the existing network of weather radars, and it can be used to alert growers that local crops are at heightened risk (Westbrook & Isard, 1999; Drake, 2002). Such information can then be used for fine tuning pest management practices such as pesticide applications, and could potentially reduce pesticide use by nearly 50% and lessen the overall impact of toxic chemicals on the environment (Dupont, Campanella & Seal, 2000), as well as on the natural enemies of these insect pests. Table 2 shows example applications of different remote sensing technologies used to detect change in vegetation.

**Table 2:** Example applications of the use of remote sensing technologies to detect change in vegetation.

Satellite and aircraft sensor	Spatial resolution	Biophysical variables for vegetation
Landsat 7 (ETM+)	15 m Panchromatic (Pan) bands; 30 m in the sex VIS, NIR, IR, and shortwave (SWIR) infrared bands; and 60 m in the thermal infrared bands	Designed to monitor seasonal and small-scale processes on a global scale such as cycles of vegetation and agriculture
Landsat 8 (OLI)	15 m pan bands; 30 m in the sex VIS, NIR, SWIR1, SWIR2; and 30 m in the cirrus bands	
ASTER	15 m in the VIS and NIR range, 30 m in the shortwave infrared band	Land cover classification and change detection
NOAA (AVHRR)	1.1 km spatial resolution	Large-area land cover and vegetation mapping
SPOT	5 and 2.5 m in single-band, and 10 m in multiband	Land cover and agricultural
GeoEye/IKONOS	Panchromatic at 1 m resolution and multispectral at 4 m resolution and colour images at 1 m	Pigments Canopy structure Biomass derive from vegetation indices Leaf index Vegetation stress Absorbed photosynthetically active radiation Evaporations
Digital Globe’s/QuickBird	Panchromatic with 61 cm resolution and multispectral images with 2.44 m resolution and colour images with 70 cm	
RADAR (SAR)	3 m resolution	
LIDAR	0.5–2 m resolution and vertical accuracy of less than 15 cm	

### Spectroscopic analysis

Fluorescence spectroscopy (FS) is a type of spectroscopic method by which fluorescence is measured of an object of interest following excitation by rays of light. Fluorescence has been used for vegetation research to monitor stress levels and physiological states in plants. There are two types of fluorescence. The first is blue-green fluorescence in the ∼400–600 nm range and the second type is chlorophyll fluorescence in the ∼650–800 nm range. Fluorescence spectroscopy can be used to monitor nutrient deficiencies, environmental conditions based on stress levels, infestations, and plant diseases. In fact, it can be used to monitor fruit quality, photosynthetic activity, tissue stress, and infestations in many types of crops (Karoui & Blecker, 2011; Tremblay, Wang & Cerovic, 2012).

Remote Sensing is a powerful technique for visualising, diagnosing, and quantifying plant responses to stress like temperature, drought, salinity, flooding, and mineral toxicity. Approaches can range from the use of simple combinations of thermal and reflectance sensor data to visible reflectance and fluorescence data. In particular, combined fluorescence reflectance and thermal imaging sensor data can be used for quick investigations of vegetation stress (Lenk et al., 2007).

### Solar radiation and the humid-thermal ratio

Biological systems are highly dependent on two most important climatic factors, namely temperature and precipitation. Temperature is influenced by solar radiation and thermal emissions, while precipitation controls the dry or wet conditions (humidity) associated with plant growth. These factors are especially important in regions where extreme temperatures and humidity conditions are prevalent and large fluctuations exist throughout the seasons as such conditions can predispose plants to insect pests and diseases. In this regard, solar radiation models can be used to investigate insect infestations. Solar radiation models can be applied to calculate the potential solar radiation at a chosen location over a 12-month period.

**Figure 2:**
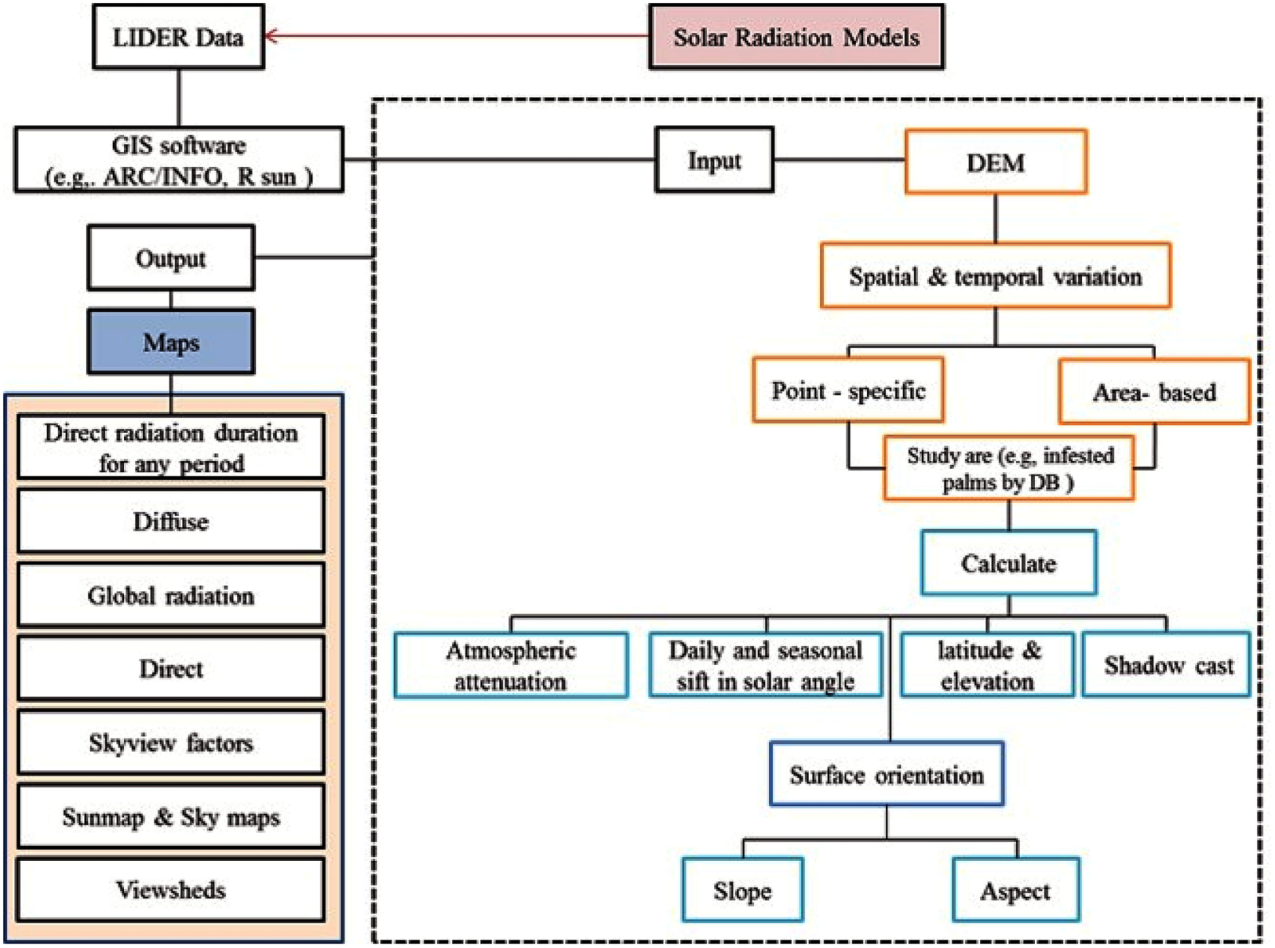
A diagram showing the design and use of solar radiation models to analyse the relationship between Dubas bug infestation levels and positional solar radiation.

The results from solar radiation studies can then be used to find correlations with different infestation levels to examine if solar radiation plays a determinant role in different infestation levels (see Fig. 2). Solar radiation can also be used to study the presence/absence and density of animals, plants diseases and infestations such as those caused by DB. More information on the theory and technical aspects of solar radiation models can be found in Bonan (1989), Dubayah & Rich (1995), Flint & Childs (1987), Geiger et al. (2002), Hetrick et al. (1993), Kumar, Skidmore & Knowles (1997), Kirkpatrick & Nunez (1980), Mazza et al. (2000), and Swift (1976).

The humid-thermal ratio (HTR) has successfully been used to develop and test relationships between different plant infestations levels in varied climate conditions in areas such as Australia, India, Europe, and North America. An HTR prototype has been developed to simulate ecological conditions appropriate for the establishment and spread of plant diseases in India (Jhorar et al., 1997). The HTR method has also been used to evaluate the risk of the establishment and spread of *Karnal* in wheat, grown under a variety of climatic conditions and in different areas (Mavi et al., 1992; Stansbury & Pretorius, 2001; Workneh et al., 2008). This method has potential value in researching insect pests and their associated diseases, which may allow for the prediction of occurrence and non-occurrence under specific combinations of climate and weather conditions.

## Vegetation

### Image processing for vegetation

**Figure 3:**
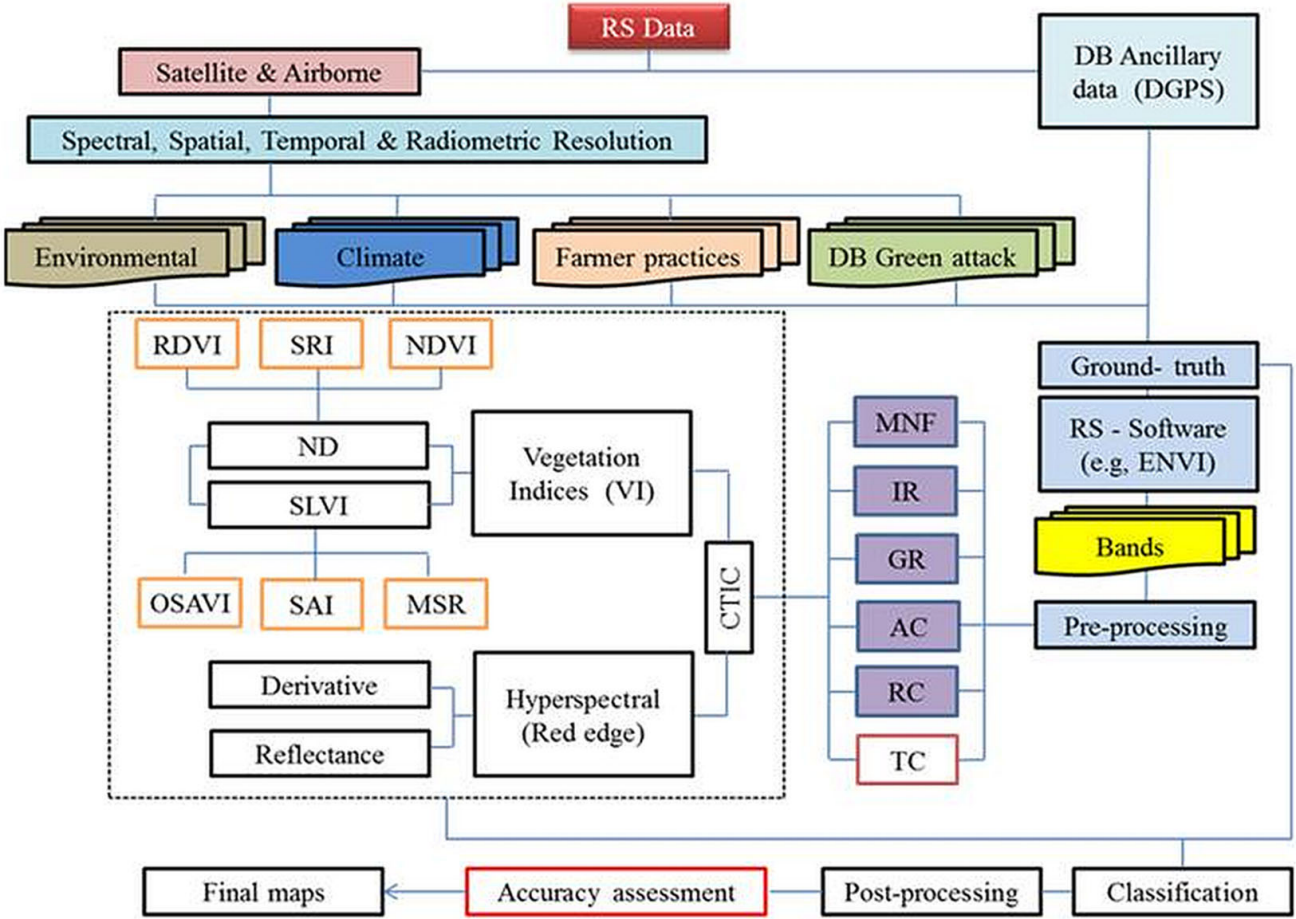
Flowchart of an image processing methodology, which include three main steps for implementing change detection research, namely: (1) image pre-processing work: geometrical replication (GR), image registration (IR), minimum nose fraction (MNF) analysis, radiometric correction (RC), atmospheric correction (AC), and topographic correction (TC); (2) selection of optimal techniques to conduct the change detection; and (3) accuracy assessments to obtain final maps.

In order to detect changes, important information must be provided including spatial distributions of change, change rates, change trajectories for different vegetation types, and assessment of the accuracy of the change detection results. The three main steps in implementing change detection are (1) image pre-processing, e.g., geometrical rectification (GR), image registration (IR), minimum noise fraction (MNF) analysis, radiometric, automorphic, and topographic correction (the latter is needed if the study area is close to mountains) (Bagheri et al., 2016; Bishop & Colby, 2002; Civco, 1989; Teillet, Guindon & Goodenough, 1982); (2) selection of optimal techniques to conduct the change detection analysis; and (3) accuracy assessments (Datt et al., 2003; Lu et al., 2004; Lunetta et al., 2006; Lyon et al., 1998; Song et al., 2001) (see Fig. 3).

Although the selection of appropriate change detection techniques is important for the accuracy of change results, in practice, it might not be easy to select a suitable algorithm for a specific change detection application. Some simple techniques can be used to provide change and non-change information (e.g., image differencing). Other techniques may be used to provide a complex matrix of change direction data such as that used for post-classification comparisons (Lu et al., 2004). This review provides examples of change detection methods that can be used to address DB infestations and their impacts on date palm trees.

### Techniques and methods

#### Vegetation indices

Vegetation indexes (VIs) are used to compile data into a single number that quantifies vegetation biomass and/or plant vigour for each pixel in a RS image. An index is computed by using several spectral bands that are sensitive to plant biomass and vigour (McFeeters, 1996). Such indices can be used to (1) specify the amount of vegetation (e.g., biomass, SAVI, the percentage of vegetation cover); (2) discriminate between soil and vegetation; and (3) reduce atmospheric and topographic effects. However, variability in VI data can arise from atmospheric effects, viewing and illumination angles, sensor calibrations, errors in geometric registration, subpixel water and clouds, snow cover, background materials, image compositing, and landscape topography (e.g., slope and relief). For example, in sparsely vegetated areas, the reflectance of soil and sand are much higher than the reflection of vegetation; so the detection of reflection from the vegetation cover is difficult.

##### Difference vegetation index

The difference vegetation index (DVI) is the simplest vegetation index (DVI = NIR–Red). DVI is sensitive to the amount of vegetation, and it can be used to distinguish between soil and vegetation. However, it does consider the difference between reflectance and radiance caused by the atmosphere and shadows (Jiang et al., 2006). Previous research (Glenn et al., 2008) that used the utility of image differencing, image rationing, and the vegetation index for detecting gypsy moth defoliation found that a difference of the MSS7/MSS5 ratio was more useful for delineating defoliated areas than any single band-pair difference.

##### Ratio-based vegetation indices

Ratio-based vegetation indices are also called the simple ratio (SR) or RVI (SR = NIR/Red). The SR provides valuable information about vegetation biomass or leaf area index (LAI) variations in high-biomass vegetation areas such as forests. It is also useful in low-biomass situations, such as those containing soil, water, ice, etc., where the SR indicates the amount of vegetation present. The SR is capable of reducing the effects of the atmosphere and topography on the analysis results.

##### Normalised difference vegetation index

Normalised difference vegetation index (NDVI) are generally well-documented, quality-controlled data sources that have been re-processed for many applications and problems. It is possible to use the NDVI values to discriminate between dense forests, non-forested areas, agricultural fields, and savannahs; however, distinguishing between forests with different dominant species is not possible by using this type of RS data because several assemblages of plant species can produce similar NDVI values or similar NDVI temporal trends. Atmospheric conditions are another aspect that must be considered when using the NDVI (Willers et al., 2012).

One study, Nageswara Rao et al. (2004), reported that bananas and coconuts have close greenness profiles in mid-April, but have rather distinct greenness profiles in mid-March. Another study Chavez & MacKinnon (1994) reported that red band image differencing provided better change detection results for vegetation than red data when using the NDVI in arid and semi-arid environments of south-western United States. The NDVI may not be appropriate to use in dry areas, and caution is warranted for such applications. Date palms trees are often planted in a regular grid pattern, as are olive trees and such trees may be able to be easily distinguished with NDVI data.

##### Normalisation difference moisture index

The normalisation difference moisture index (NDMI) data can be used to determine the threshold presence of pest infestations (green attack). Such data can also be potentially used for deriving regional estimates of the year of stand death, for example, by using Landsat data and decision tree analysis. However, there are limitations associated with using the NDMI, which include difficulties in detecting low rates of infestation and the need to add images from other dates (to achieve a higher temporal frequency) to quantify the spectral response to insects such as the DB.

The application of a VI such as the NDVI and SAVI to multispectral satellite imagery (blue, red, and NIR) has been shown to be useful to quantify variations in plant vigour, make relative biomass predictions, assess yields and investigate the occurrences of pests and disease attacks outbreaks (Plant, 2001). Landsat TM data can be used to assess both plant age and LAI values by applying a number of indices such as the shadow index (SI), bare soil index (BI), NDVI, and advanced vegetation index (AVI).

#### Transformation

Feature space transformation, which relates to band space, involves processing data that are *n*-dimensions. It may be difficult to visualise these data because the feature space (where *n* is roughly the number of bands). However, several mathematical techniques are readily available to analyse the feature space; they include principal components analysis (PCA), Kauth’s Tasseled Cap (KTC), perpendicular vegetation index (PVI), leaf water content index (LWCI), SAVI, NDMI, atmospherically resistant vegetation index (ARVI), aerosol free vegetation index (AFRI), global environmental monitoring index (GEMI), and red-edge position (REP) determination (Eitel et al., 2011). These techniques and many more can be used to find areas that contain plentiful spectral information.

The PCA and the KTC transformations can be used for land cover change detection via NIR reflectance or greenness data that can detect crop type changes between vegetation and non-vegetation features (Gorczyca, Gong & Darzynkiewicz, 1993; Lu et al., 2004). An earlier study (Rondeaux, Steven & Baret, 1996) found that SAVI, where the value *X* was tuned to 0.16, easily out-performed all other indices when applied to agricultural surfaces. Others (Kaufman & Tanre, 1992; Leprieur, Kerr & Pichon, 1996) have concluded that the GEMI and ARVI are less sensitive to atmosphere, but may be incapable of dealing with variation in soil reflectance. More information about feature space transformation can be found in Gebauer et al. (2007) and Luedeling & Buerkert (2008). According to Darvishzadeh et al. (2008), REP is the most studied feature on vegetation spectral curve because it is strongly correlated with foliar chlorophyll content and can be a sensitive indicator of stress in vegetation.

#### Classification

The objective of image classification is to categorise all pixels in the imagery into one of several land cover classes or themes. The categorised data can then be used to produce thematic maps of land cover (e.g., vegetation type) based on remotely sensed data. Most image processing techniques offers several methods to test hypotheses. The best-known methods include supervised and unsupervised classification; however, these techniques require ground reference data.

Maximum likelihood classification, for example, requires samples of pixels obtained by field observations or aerial photography interpretations that are deemed to be representative of specific land cover types. The maximum likelihood method relies on the assumption that the populations from which these training samples are drawn are multivariate–normal in their distributions. The traditional methods employ classical image classification algorithms (e.g., *k*-means and ISODATA) for unsupervised classification, and maximum likelihood classification for supervised classification.

##### Maximum likelihood classification algorithm

The maximum likelihood classification algorithm (or parametric information extraction) is the most widely adopted parametric classification algorithm. However, it requires normally distributed training data, especially for *n* (rarely the case) to compute the class variance and covariance matrices. Another limitation is that it is difficult to integrate non-image categorical data into a maximum likelihood classification. However, fuzzy maximum likelihood classification algorithms are also available (Zhang & Foody, 2001).

##### Classification techniques

*Supervised classification.* The supervised classification methods can be used to select representative samples for each land cover class in a digital image. Sample land classes are more commonly called training sites. The image classification software uses the training sites to identify the land cover classes in the entire image. The classification of land cover is based on spectral signatures defined in the training set. The digital image classification software determines the class based on what it resembles most in the training set. The limitation on the use of supervised classification is that analysis is required to identify areas on an image of known informational types and to create a training area (group of pixels) from which the computer generates a statistics file (Mountrakis, Im & Ogole, 2011).

*Unsupervised classification*. The advantage of the use of unsupervised classification is that all spectral variation in the image are captured and used to group the imagery data into clusters. The major disadvantage is that it is difficult to completely label all the clusters to produce the thematic map.

*Combined and advanced methods.* Many examples exist whereby the supervised and unsupervised techniques were combined together in analyses. The associated advantages and disadvantages can be found in Castellana, D’Addabbo & Pasquariello (2007) and Pao & Sobajic (1992). However, the combined approach only slightly improves the ability to create thematic maps when compared to using each technique separately. Moreover, a large amount of effort has been devoted to developing advanced classification approaches to improve our ability to create thematic maps from digital remotely sensed imagery. One of the most recent advances has been the adoption of artificial neural networks (ANNs) in the place of maximum likelihood classification (standard in most RS software). This review only covers a few of the non-parametric techniques.

*Artificial neural network*. Fortunately, the ANN methods (non-parametric information extraction) do not require normally distributed training data, and may be used to integrate with virtually any type of spatially distributed data in classification. The disadvantage of using ANN is that occasionally it is difficult to determine exactly how the ANN came up with a certain assumption because such information is locked within weights in a hidden layer or layers. The method has been used successfully for classifying infestations, diseases/conditions of plants and the associated damage based on spectral data (Cox, 2002; Liu, Wu & Huang, 2010; Pydipati, Burks & Lee, 2005). In recent years, spectral mixture analysis, ANNs, GISs, and RS data have become important tools for change detection applications.

*Artificial intelligence.* Use of nonmetric information extraction or AI methods allows the computer to analyse data perhaps better than people. The benefits of using AI for image analysis involve the use of expert systems that place all the information contained within an image in its proper context with ancillary data and then to extract valuable information (Duda, Hart & Stork, 2001).

*Classification and regression tree*. Classification and regression tree is a non-parametric algorithm that uses a set of training data to develop a hierarchical decision tree. The decision tree is created by using a binary partitioning algorithm that selects the best variable by which to split the data into separate categories at each level of the hierarchy. Once the final tree is generated, it can be used to label all unknown pixels in the image. This method is extremely robust and provides significantly better map accuracies than those that have been achieved by using more basic approaches (Lawrence & Wright, 2001).

*Support vector machines.* Support vector machines are derived from the field of statistical learning theory and have been used in the machine vision field for the last 10 years. These methods have been developed for use in creating thematic maps from remotely sensed imagery. The SVM performs by projecting the training data using a kernel function and this results in a data set that can then be linearly separated. The capability to separate out the various informational classes in the imagery is a powerful advantage. The use of SVM is relatively new, but it offers great potential for creating thematic maps from digital imagery.

Several advanced techniques for classifying digital remotely sensed data involve the extensive development and adoption of object-based image analysis (OBIA). Moreover, advanced image classification techniques such as *k*-means, ISODATA, fuzzy ARTMP, fuzzy multivariate cluster analysis, the WARD minimum variance technique, SOM, the artificial neural classification algorithm (i.e., for the propagation of neural networks and self-organising maps) and Bayesian analysis can be used (1) for the classification of remotely sensed data; and (2) to delineate horticultural crops in satellite maps. The major advantage of these techniques is their ability to generate a matrix of change information and to reduce external impacts from the atmospheric and environmental differences among the multi-temporal images. However, it may be difficult to select high quality and sufficiently numerous training sets for image classification, in particular for important historical image data classifications due to the lack of data (Lu, Moran & Batistella, 2003; Lu & Weng, 2007; Lunetta et al., 2006; Monteiro, Souza & Barreto, 2003; Rogan, Franklin & Roberts, 2002).

All these classifications are performed on a pixel-by-pixel basis. Therefore, given that a pixel maps an arbitrary delineation of an area on the ground, any selected pixel may or may not be representative of the vegetation/land cover of that area. In OBIA, unlabelled pixels are grouped into meaningful polygons that are then classified as polygon pixels (Blaschke, 2010; Dey, Zhang & Zhong, 2010; Haralick & Shapiro, 1985; Stafford, 2000).

Classified satellite imagery can also be used to extract palm crown data. The centre of crowns can be isolated because they often remain green and are not as severely impacted by the DB as the palm fronds. Densities of the DB tend to be highest outside of the crown region. The removal of the centre and concentration on the outer parts of the vegetation can then lead to a higher probability of detecting the impacts of DB and categorising the infestation levels accurately. The images can also be used by classification techniques (e.g., unsupervised) to detect stages for which users do not have ground truth data.

#### Image segmentation techniques

Image segmentation techniques can be used to extract information on palm canopies. The crown information can be used to calculate the density of palms per unit. This information can then be applied as part of a GIS-based spatial analysis to answer questions about whether infestation levels are linked to the density of palms or not. The crown information could also be used to determine the random or systematic nature of farms.

This information can be further used in GIS-based analyses to answer questions about whether or not randomly situated plants have a higher risk of infestation than non-randomly situated plants. Such information would be useful for determining the optimal row spacing. Research published in the literature suggests that those plantations that have wide row spacing have a lesser likelihood of DB infestations (Ali & Hama, 2016). The row spacing data extracted from satellite imagery could thus be used to confirm the relationship between row spacing and infestation levels.

#### Image fusion

Image fusion is a technology that merges two or more images of the same area collected by different sensors or at different wavelengths. For example, merging a 2.5 m multispectral image with a 0.7 m panchromatic image can be done to capitalise on the advantages of both image sets. The panchromatic images have very good spatial resolution but lack the multiband information that the 2.3 m multispectral image provides. Thus, the advantage of using image fusion for change detection is that fusion can allow for both high spatial and spectral resolutions, which will enable users to extract high quality land cover/vegetation information (Boryan et al., 2011; Simone et al., 2002). Image fusion techniques such as the HSV (hue, saturation, value), Brovey, Gram-Schmidt, and principle components methods can be used to compare the accuracy and distortion levels of images (e.g., 8-band Worldview images).

## Accuracy Assessment

Accuracy assessment is an important part of any classification algorithm process, and it should be undertaken for every project because it is difficult to know how accurate a classification is without an accuracy assessment. The accuracy of a classification is usually assessed by comparing the classification with some reference data that is believed to accurately reflect the true land-cover. Reference data may include ground truth data, higher resolution satellite images and maps derived from aerial photographic interpretations. However, in the case for all reference data, even ground truth data, these data sets may also contain some inaccuracies. More information about accuracy assessments can be found in Al-Kindi et al. (2017b), Congalton (2001), Foody (2002), Gibbs et al. (2010), Hirano, Welch & Lang (2003), Huang et al. (2007), and Hughes, McDowell & Marcus (2006).

Positional accuracy methods can be used to provide an assessment of the differences in distance among a sample of locations on the map and those same locations on a reference data set. This same basic process can be used in assessing the thematic accuracy of a map, and it involves a number of initial considerations such as taking into account the sources of errors and the proper selection of classification systems (Congalton & Green, 2008). Determination of the thematic accuracy is more complicated than that of the positional accuracy.

This is due to the size requirements for sampling thematic accuracy assessments, which are larger than those for positional accuracy assessments. An error matrix technique can be used to compute the thematic accuracy, and the error matrix can be generated by using reference data and correct or incorrect designations; one can also use qualifiers such as good, acceptable and poor to produce a fuzzy error matrix. Additionally, there are a number of analysis techniques that can be performed using the error matrix, such as the Kappa analysis. The Kappa analysis can be used to test statistically whether or not one error matrix is significantly different than another (Goodchild, 1994).

## Modelling the Spatial Relationships Between Insect Infestations and the Environmental and Climate Factors

While RS techniques focus on visual and pre-visual detection and mapping, spatial analytical techniques can be used to evaluate correlations, identify important variables, and develop predictive models. Spatial statistics functions and tools have made it possible to implement state-of-the-art spatial autoregressive techniques to investigate many research problems (e.g., insect pest) (Carrière et al., 2006; Carruthers, 2003; Wulder et al., 2006). Advances in spatial analytical techniques software, such as ArcInfo®, have greatly reduced the time for estimating spatial parameters. For example, regression analysis allows users to examine, model, and explore spatial relationships in order to better understand the factors behind the observed spatial patterns. It also allows users to predict hypotheses based on understanding of these factors. There are three main types of regressions, namely, linear regression, local regression, and logistic regression (Liebhold, Rossi & Kemp, 1993; Wichmann & Ravn, 2001). Linear regression can be used to predict the values of *y* from values of *x_i_* as follows:

(1) $$y = a + {b_1}{x_1} + {b_2}{x_2} + ... + {b_n}{x_n}$$

where *y* is the dependent variable, *x_i_* represents the independent variables *i*, and *b_i_*, …, *bn* are the regression coefficients. However, this requires several assumptions about the error, or residuals, between the predicted values and the actual values (Miles & Shevlin, 2001). Some errors are related to a normal distribution for a set of independent variables, while others are related to the expected mean value of zero. Linear regression has been used to model wildlife home ranges (Anderson et al., 2005) and soil moisture (Lookingbill & Urban, 2004; Lema, Mendez & Blazquez, 1988). According to Harris et al. (2010), local regression or geographically weighted regression (GWR) analysis can be used to predict information for every known point in order to derive a local model. Moreover, parameters for this method can include variations in space, thereby providing a basis for exploring non-stationary spatial relationships. The logistic regression method can be applied to model spatial relationships between features, such as when the dependent variable is categorical (e.g., presence or absence data) and when the independent variables are categorical, numeric, or both (Menard, 2002). The advantage of using the logistic regression is that it does not require the same set of rigid assumptions as required by linear regression.

Various studies have involved the use of autoregressive models to investigate the relationships between insect infestations and factors that are based on environmental information. Munar-Vivas, Morales-Osorio & Castañeda-Sánchez (2010) combined environmental information, spatial data, and attribute data in GIS-based maps to assess the impact of *Moko* disease on banana yields in Colombia. Specifically, they used a regression model to investigate the relationship between infested areas and distances from the *Moko* foci to cable-ways and drainage channels. Coops et al. (2006) studied the associations among the likelihood of occurrence, forest structure and forest predisposition variables using regression tree models. They found through modelling that location and slope were the major factors driving variations in the probability of red tree outbreaks. The GWR model has been used to detect high-risk infestations caused by mountain pine beetle invasions of lodge-pole pine forests over large areas (Robertson et al., 2008).

It is important to start by using single variables to develop correlations before moving to more complicated predictive models and regression analyses, where all factors are incorporated to investigate which combination of factors is most conducive to the survival and spread of insects or diseases. In our study, for instance, GWR could be used to model the correlation between DB infestation and meteorological variables such as humidity, rainfall, temperature, wind direction, and wind speed; GWR could also be applied to model the correlations between DB infestations and environmental variables including soil type, slope, aspect ratio, ecology, soil salinity, and solar radiation. Additionally, autoregressive models could be used to investigate the relationships between DB infestations and human practices such as irrigation, plantation systems, insecticide use, and methods of spraying (Al-Kindi et al., 2017a).

### Suitability model for detecting and investigating insect infestations

All of the methods used to study the relationships between dependent and independent variables discussed previously are traditional statistical methods, which sometimes might not reflect the complicated relationships between infestations and environmental factors. In particular, ecological and geographical environments represent complex systems in which individual elements interact to create complex behaviour, and consequently, complex methods such as ANN, Cellular Automata (CA), and multi-agent systems (MAS) may be better suited to study the relationships and conduct factor analyses in insect infestation or disease detection research and to perform spread simulations (De Smith, Goodchild & Longley, 2007).

Numerous suitability models have been proposed to identify locations that have a particular set of characteristics.

In Hernandez et al. (2006), the authors compared four different models (BIOCLIM, GAPP, DOMIN, and MAXENT) and found that MAXENT was most capable for producing useful results with small sample sizes and minimum species occurrences. These models can also be used to identify areas that are susceptible to risks such as insect infestations, based on conditions favoured by the species. For example, a relevant study (Drees et al., 2010) used the habitat suitability selection method to model potential conservation areas for a rare ground beetle species (using barcode index number or BIN). Specifically, they used five different data sets to identify several key habitat factors for *Carabus variolosus* stress levels. A model was developed in Bone, Dragicevic & Roberts (2005) by using fuzzy theory to identify areas of susceptibility to *Dendroctonus ponderosae* Hopkins in Canada. However, spatial data have unique characteristics that can impact the results of the model (Crooks & Castle, 2012).

**Figure 4:**
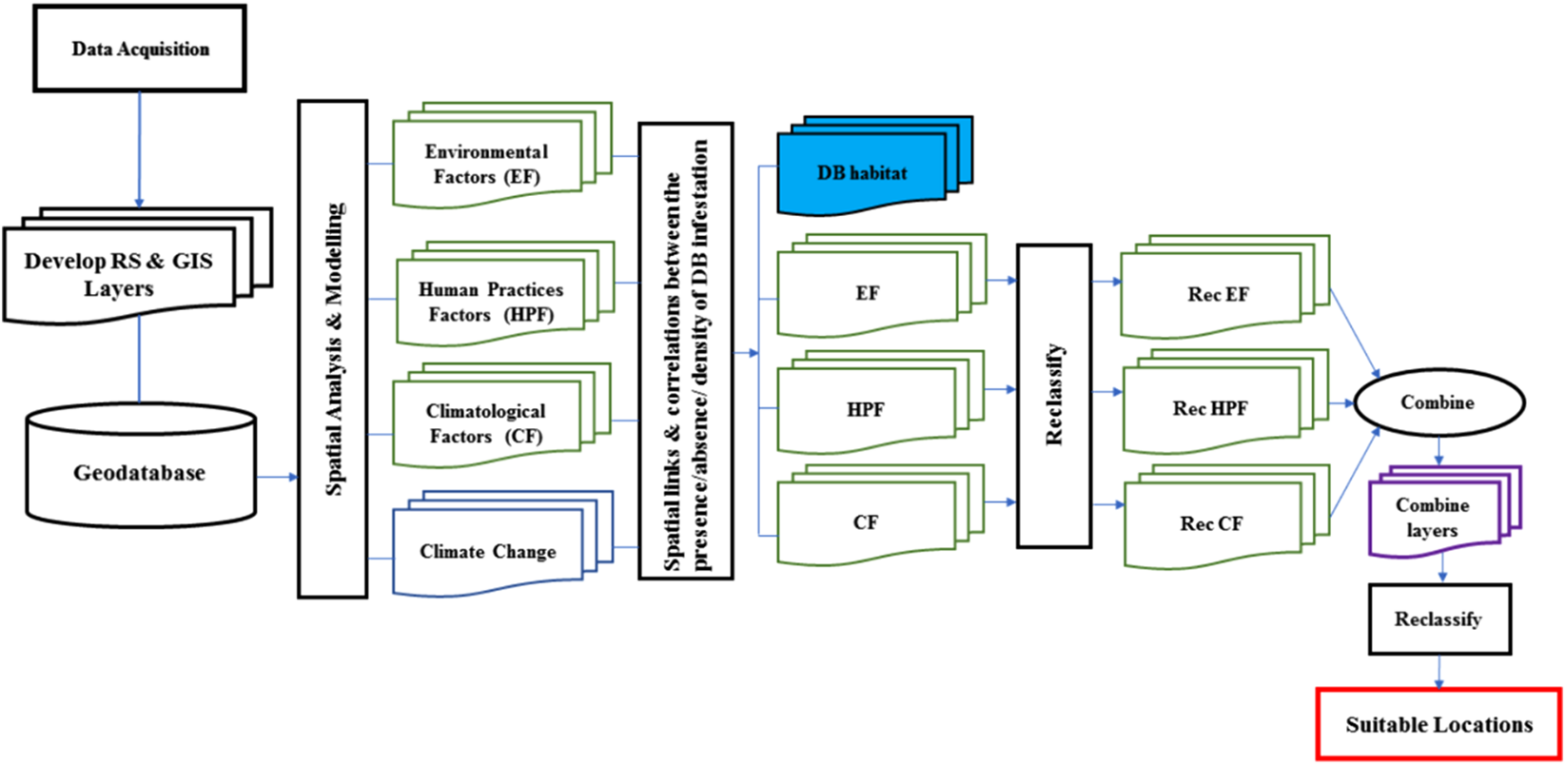
Schematic of the process that can be used to model the suitable location for Dubas bug infestations.

Raster data models are often used for finding and rating suitable locations. The raster overlay results are formatted in a single layer of suitable versus unsuitable cells, rather than in a vector layer with many polygons and an attribute table, which contains the attribute values for each of the polygons. There are two ways to create raster suitability layers. The first approach is to query the individual sources to create the suitability layer. The query can be used to create a suitability layer with two values, ‘1’ for cells meeting all criteria of a suitable habitat, and ‘0’ for the others. Because the layer consists of only two values, one indicating suitable and the other unsuitable cells, they are called binary suitability layers. Binary processing however is not always necessary. Combined with other evaluation models, suitability mapping can be achieved by overlaying directly or by post processing the overlay results. Figure 4 shows a process that could be used to find suitable location conditions (habitat) for insects such as DB by using a raster method overlay.

The uncertainty that results from geo-processing operations, demonstrates that sophisticated spatial analysis cannot be achieved using traditional, deterministic geoprocessing methods alone (Goodchild & Glennon, 2010; Zhang & Goodchild, 2002). Fuzzy logic is a superset of Boolean logic and has the ability to handle uncertainty in data that arises from vagueness instead of randomness alone (Li et al., 2010).

Fuzzy logic can be utilised to extract information from high resolution RS data and combined with a raster-based spatial data to produce maps representing the spatial variation of vulnerability to pests across a landscape (Zhang & Foody, 2001). This method also allows for partial association with one or more classes, meaning that objects may be represented by a value based on a membership function between ‘0’ and ‘1’ (Li & Zhao, 2007). The membership function of an element *x* belonging to a fuzzy set A is computed by:

(2) $${{\rm{\mu }}_{\rm{A}}}:U \to [0,1]$$

where *U* is the universal set of *x*. The concept of fuzzy sets has also been employed for defining the spatial and attributes characteristics of geographic objects (Burrough & Frank, 1996; Wang & Hall, 1996). The results of such analysis can be rendered directly into a decision framework via maps, tables, and charts. The results can also be used in further analyses or to provide additional understanding of the problem.

The challenge in any particular area of study is the geographical extent and the resolution of analysis, which is determined by the phenomenon being modelled. To achieve validity, researchers must ensure that they are using accurate and current data whenever possible. If the data are from one’s own organisation, one can rely on data quality controls that are in place. Data quality should be checked against alternate sources if possible in order to ensure it meets the requirements of the analysis. Assessing the quality of data will provide guidance to predicting what level of confidence can be attributed to the result of the modelling work.

## Proof-of-Concept Cases

The first proof-of-concept case is published in Al-Kindi et al. (2017a). In this paper, we analysed a set of IKONOS satellite images collected in 2015 on our study area (5 m spatial resolution) by processing them using chosen image segmentation functions and extracted density information of the palm canopies. The techniques used can be found in ‘Image Segmentation Techniques.’

Next, sample locations (i.e., GPS points) were identified in the satellite images by examining their normalised different vegetation index (NDVI) values. NDVI served as a surrogate measure of palm plantation density and homogeneity in the neighbourhood surrounding an image pixel. The relevant techniques can be found in ‘Normalised Difference Vegetation Index.’

In addition, spatial statistical techniques including GWR, Ordinary Least Squares and Exploratory Regression (corresponding implementations included in ArcGIS™) were applied to study the correlations between various human factors related to date palm farming and the distribution density of the DB. These techniques have been reviewed in ‘Modelling the Spatial Relationships between Insect Infestations and the Environmental and Climate Factors.’

The second proof-of-concept case is published in Al-Kindi et al. (2017b). In that paper, we applied spatial statistical techniques to model spatiotemporal patterns of DB on date palm in north of Oman. Data on the DB infestations and their impact were collected through observations of palm trees from 2006 to 2015 by the Ministry of Agriculture and Fisheries of the Sultanate of Oman. The techniques used can be found in ‘Modelling the Spatial Relationships between Insect Infestations and the Environmental and Climate Factors’ and ‘Data Requirements for Crop Management.’

## Conclusion

In this review, a variety of spatial information technologies, including remote sensing and spatial statistical methods, have been shown to be useful in areas of research involving insect infestations worldwide. Environmental and climatic conditions are very important in determining the distribution and survival of any species, including the DB, which is a problematic pest in date palm plantations. We argue that most of the current research on DB has focused on its ecology, biology, or control mechanisms only. There has been very limited research linking the presence/absence, density, spatial, and temporal distributions of DB with environmental, meteorological, and human practices that promote its development, prevalence, and spread. Understanding the distribution and affinity of the DB in terms of these variables and mapping of the data can play a key role in its control and management, as well as resource allocation.

## Abbreviations

AFRI: aerosol free vegetation index
ANN: artificial neural network
AI: artificial intelligence
ASTER: advanced space thermal emission and reflection radiometer
AVHRR: advanced very high resolution radiometer
AVIRIS: airborne visible/infrared imaging spectrometer
ALOS: advanced land observing satellite
AC: atmospheric correction
ARVI: atmospherically resistant vegetation index
BIO: bare soil index
CA: cellular automata
CART: classification and regression tree
CIR: colour-infrared
DEM: digital elevation model
DB: Dubas bug
DVI: different vegetation index
NDV: normalised different vegetation
NDMI: normalisation different moisture index
FS: fluorescence spectroscopy
GIS: geographical information systems
GEMI: global environmental monitoring index
GR: geometrical rectification
GWR: geographically weighted regression
HTR: humid-thermal ratio
IPM: integrated pest management
IR: image registration
LIDAR: light detection and ranging
LAI: leaf area index
LWCI: leaf water content index
MIR: mid-infrared
MODIS: moderate resolution imaging spectroradiometer
MAS: multi-agent system
MNF: minimum noise fraction
MSS: multi-spectral scanner
NAIP: national agricultural imagery programme
NIR: near-infrared
OBIA: object-based image analysis
PCA: principal components analysis
PVI: perpendicular vegetation index
REPD: red-edge position determination
RS: remote sensing
RVI: ratio vegetation Index
SAVI: soil adjusted vegetation
SCI: shadow canopy index
SPOT: Satellite Probatoire l’Observation de la Terre
SVM: support vector machines
TM: thematic mapper
TC: topographic correction
UAV: unmanned aerial vehicle
VIS: visible
VI: vegetation indices

## Additional Information and Declarations

### Data Availability

The following information was supplied regarding data availability: The research in this article did not generate any data or code (literature review).
